# Calidad de vida de estudiantes de la Facultad de Medicina de la Universidad Nacional de Córdoba

**DOI:** 10.31053/1853.0605.v80.n3.41147

**Published:** 2023-09-29

**Authors:** Santiago Romero, Matías Agustín Regis, Elena María Rosa Díaz, Guillermo Einer Allende, Gustavo Alberto Pepe, Carla Andrea Gobbi

**Affiliations:** 1 Universidad Nacional de Córdoba. Facultad de Ciencias Médicas. Cátedra de Clínica Médica I. Hospital Córdoba, UHMI N°3.

**Keywords:** calidad de vida, estudiantes de medicina, educación médica, quality of life, students, medical, education, medical, qualidade de vida, estudantes de medicina, educação médica

## Abstract

**Introducción:**

La Calidad de Vida (CV) pobre, puede interferir en los procesos de enseñanza aprendizaje.

**Objetivo:**

Describir la CV de estudiantes de medicina de la Universidad Nacional de Córdoba (UNC), comparando los Estudiantes Iniciales (EI) de los primeros tres años versus los Estudiantes Avanzados (EA), segundos tres. Métodos: Estudio transversal, observacional y analítico, utilizando cuestionario WHOQoL-BREF enviado mediante el sistema Guaraní de la UNC. Se valora la CV global y satisfacción respecto al estado de salud, con cuatro dominios físico, psicológico, relaciones interpersonales y entorno. p<0.05 fue significativa. El trabajo fue aprobado por el CIEIS del Adulto.

**Resultados:**

Se obtuvieron 854 respuestas, el 72% fueron mujeres, el 43% fue originario de otra provincia/país y el 63% eran EI. Los EI presentaron puntajes más altos en CV bastante buena y muy buena OR 1.65 (1.23-2.2) con una p<0.00058, al considerar todos los dominios no hubo diferencia entre los grupos. La actividad física en ambos grupos se relaciona con los mejores valores de CV, en el grupo de EI OR 1,95 (1,31-2,92) p <0,0009; mientras que en EA, OR 4,13 (2,49-6,87) p <0,00000001. El consumo de alcohol fue alto, siendo mayor en los EI al igual que el tabaco, mientras el consumo de marihuana y modafinilo en los EA. No hubo diferencia en cuanto al sexo y procedencia en CV. El alfa de Cronbach fue 0.8899.

**Conclusión:**

La CV es mejor percibida en el grupo de estudiantes iniciales y en quienes realizan actividad física.

## Abstract (en)

**Introduction:**

Poor Quality of Life (QOL) can interfere in the teaching-learning processes.

**Objective:**

To describe the CV of medical students at the National University of Córdoba (NUC), comparing the Initial Students (IS) of the first three years versus the Advanced Students (AS), second three. Methods: Cross-sectional, observational, and analytical study, using the WHOQoL-BREF questionnaire sent through the Guarani system of the NUC. Global QoL and satisfaction with the state of health are assessed, with four domains: physical, psychological, interpersonal relationships and environment. p<0.05 was significant. The project was approved by the Adult CIEIS.

**Results:**

854 responses were obtained, 72% were women, 43% were from another province/country and 63% were IS. The IS presented higher scores in good and very good QOL questions OR 1.65 (1.23-2.2) p<0.00058, when considering all the domains there was no difference between groups. Physical activity in both groups is related to the best QoL values, in the IS group OR 1.95 (1.31-2.92) p <0.0009; while, in AS, OR 4.13 (2.49-6.87) p <0.00000001. The consumption of alcohol was high, being higher in the IS as well as tobacco, while the consumption of marijuana and modafinil in the AS. There was no difference in terms of sex and origin in QoL. Cronbach's alpha was 0.8899.

**Conclusion:**

The QoL is better perceived in the group of initial students and in those who perform physical activity.

## Resumo

**Introdução:**

A má qualidade de vida (QV) pode interferir nos processos de ensino-aprendizagem.

**Objetivo:**

Descrever o currículo dos estudantes de medicina da Universidade Nacional de Córdoba (UNC), comparando os Alunos Iniciais (AI) dos três primeiros anos com os Alunos Avançados (AA), dos últimos anos. Métodos: Estudo transversal, observacional e analítico, utilizando o questionário WHOQoL-bref enviado pelo sistema Guarani da UNC. Avalia-se a QV global e a satisfação com o estado de saúde, com quatro domínios: físico, psicológico, relações interpessoais e meio ambiente p<0,05 foi significativo. O trabalho foi aprovado pelo CIEIS Adulto.

**Resultados:**

Foram obtidas 854 respostas, 72% eram mulheres, 43% eram de outra província/país e 63% eram AI. O AI apresentou maiores escores em QV razoavelmente bom e muito bom OR 1,65 (1,23-2,2) com p<0,00058, considerando todos os domínios não houve diferença entre os grupos. A atividade física em ambos os grupos está relacionada com os melhores valores de QV, no grupo AI OR 1,95 (1,31-2,92) p <0,0009; enquanto, em AA, OR 4,13 (2,49-6,87) p <0,00000001. O consumo de álcool foi elevado, sendo maior no AI assim como o tabaco, enquanto o consumo de maconha e modafinil no AA. Não houve diferença quanto ao sexo e procedência no CV. O alfa de Cronbach foi de 0,8899.

**Conclusão:**

A QV é melhor percebida no grupo de alunos iniciais e naqueles que realizam atividade física.

**CONCEPTOS CLAVE**

La calidad de vida es un factor importante en el rendimiento académico de los alumnos universitarios. Conocer la calidad de vida es la base para diseñar políticas universitarias acordes a la realidad del alumnado.

**Divulgación**

El estudiante de Medicina, a medida que avanza en su carrera está sometido a mayores exigencias y estrés, debido a la mayor carga horaria y el aumento de responsabilidades que implica la cualidad de ser médico, lo cual puede afectar la calidad de vida de la persona y por ende dificultar el proceso enseñanza aprendizaje. Estudiamos la calidad de vida de los estudiantes de Medicina de la Universidad Nacional de Córdoba a los fines de evaluar su estado, identificar a los grupos de riesgo que perciben una mala calidad que puede llevar a depresión y hasta suicidio con el objeto de facilitar el diseño de políticas adecuadas de prevención.

## Introducción

El concepto de calidad de vida (CV) se considera una herramienta útil para evaluar la salud general. Según la OMS, comprende la percepción que tiene una persona sobre su salud física, estado fisiológico, nivel de independencia, sus relaciones sociales, su posición en la vida dentro del contexto cultural, incluyendo el sistema de valores considerando a sus metas, expectativas, estándares y preocupaciones ^1 2^ . Gill y Feinstein sugieren que, al hablar de CV, se tiene que dar importancia a las cosas relativas a las que las personas dan cierto valor en su vida, y su origen debe ser siempre del mismo individuo ^3^ . Cada dimensión de este concepto se puede dividir, a su vez, en dos: análisis objetivo de un estado de salud y las percepciones subjetivas de la misma ^4^ . Se puede afirmar, por ende, que dos personas con el mismo estado de salud pueden tener una CV muy diferente ^5^ . La etapa universitaria es un período de transición que abarca desde la adolescencia hasta la edad adulta, durante la misma el individuo forma los hábitos que tendrá en la vida adulta ^6^ .

La calidad de vida considera dimensiones tales como: la salud física, la salud psicológica (o mental) y el contexto ambiental (o entorno social), incluyendo las relaciones sociales-interpersonales. Netuveli y Blane, definen la salud física como un estado en que el conjunto de los sistemas orgánicos que constituyen a un ser vivo ejerce normalmente todas sus funciones. En tanto, la salud mental puede ser entendida como un estado de bienestar en que el individuo es consciente de sus propias capacidades, puede afrontar las tensiones normales de la vida, puede trabajar de forma productiva y fructífera, y es capaz de hacer una contribución a su comunidad. Finalmente, el entorno social corresponde al tipo de interacción que establece un sujeto social con otro(s), según ciertas características del contexto y de los efectos percibidos sobre este, dependiendo de los roles y actividades desarrolladas ^7 8^ .

En la medida que el estudiante avanza en la carrera de medicina, se enfrenta con un alto nivel de competitividad, con una forzada y no capacitada tentativa de adaptación ambiental y muchas veces cultural, con poca vinculación social y con una desconocida y nueva responsabilidad por la vida del otro, lo cual puede originar síntomas como ansiedad, estrés, tristeza, baja autoestima, falta de motivación y concentración que deterioran la salud mental. Se enfatiza que cuando tales aspectos no son tratados o son enmascarados, pueden transformarse en depresión, ansiedad generalizada, dependencia química, burnout, y hasta suicidio ^9^ .

Sumado a lo descripto, la enfermedad por coronavirus de 2019 (COVID-19) ha mermado la salud, la economía y la vida cotidiana de millones de personas en todo el mundo. Sin embargo, podría estar afectando de manera particular a los estudiantes de Medicina, quienes han tenido dificultades para sus prácticas clínicas y ven cómo sus proyecciones futuras profesionales y laborales pueden estar cambiando drásticamente ^10^ . Considerando el plan de estudio de la Facultad de Medicina de la Universidad Nacional de Córdoba ^11^ , cuyo número de materias aumenta a medida que se progresa en la currícula, desde un promedio de 5 materias en los primeros tres años a 11 en los segundos tres, se generan en los años más avanzados, mayores obligaciones que deben afrontar los estudiantes, por lo cual es probable que la calidad de vida de los últimos años de la carrera se vea deteriorada.

La Universidad Nacional de Córdoba es una de las más importantes en nuestro país y en la misma no hay estudios previos de calidad de vida pospandemia Covid 19. Nos planteamos como objetivo conocer la calidad de vida de los estudiantes de medicina de la Universidad Nacional de Córdoba en la actualidad y comparar los tres primeros años de formación con los segundos, y como objetivos específicos determinar si los hábitos tóxicos y la actividad física influyen en la calidad de vida de los estudiantes. Además de correlacionar si algún aspecto en particular de los investigados era relevante en la población general de estudiantes.

## Material y Métodos

El estudio es de tipo descriptivo, observacional, de corte transversal, analítico.

Se invitó a participar a todos los estudiantes de Medicina de la Universidad Nacional de Córdoba durante el año 2022. El criterio de inclusión fue haber aprobado el ciclo de nivelación, aceptar formar parte del estudio y presentar regularidad de la carrera. Se excluyeron los cuestionarios que se completaron menos del 80% de las respuestas. Se envió una encuesta online a través del Sistema Guaraní, con respuesta anónima. Se evaluaron los datos sociodemográficos (edad y sexo), consumo de alcohol y drogas región geográfica de origen y actividad física, mediante respuesta dicotómica. La CV se midió a través del cuestionario WHOQoL-BREF ^12^ . Se realizó el cálculo del tamaño muestral para los estudiantes de medicina de la UNC siendo necesario un n de 362. Solo los alumnos regulares de la carrera Medicina de la Facultad de Ciencias Médicas de la UNC recibieron el cuestionario, lo respondieron a voluntad, por su propio consentimiento. La distribución de las respuestas coincide con el género del alumnado ^13^ , la regularidad en la carrera se la da el propio sistema Guaraní, al elegir a quién enviar el cuestionario. Para el análisis de datos se agruparon a los estudiantes en dos etapas de formación según su avance en la carrera: Estudiantes iniciales (EI) (1ero a 3er año) y Estudiantes avanzados (EA) (4to y 5to año). Este trabajo fue aprobado por el CIEIS del Adulto, Hospital Córdoba.

Instrumento WHOQoL-BREF: La Organización Mundial de la Salud autorizó y facilitó el instrumento en español, el cual consiste en 26 preguntas. Las dos primeras evalúan CV global y satisfacción respecto al estado de salud y las 24 siguientes evalúan cuatro dimensiones: Salud Física, Salud Psicológica, Relaciones Interpersonales y Entorno. Las respuestas son de tipo escala de Likert, con 5 opciones. En las preguntas "dolor", "dependencia a medicamentos" y "sentimientos negativos" se realizó una inversión de la escala numérica para poder ser comparadas. El cuestionario fue validado al español en dos estudios previos realizados por Espinoza y Lucas Carrasco ^14-15^ . Para su análisis en Excel se usó el método de Pedroso ^16-17^

### Análisis estadístico:

El procesamiento de la información se realizó en una base de datos Excel, se utilizaron medidas de frecuencia (absolutas y relativas) y medidas de resumen (media y desviación estándar). Se utilizó la prueba de Kolmogorov-Smirnov para determinar la distribución de los datos cuantitativos.

Para comparar una variable cuantitativa en dos grupos, se utilizó la prueba de Ude Mann Whitney. Para comparar dos variables cualitativas, se utilizó el test exacto de Fisher.

Se realizó un análisis de fiablidad global y por dimensiones del WOQoL-BREF mediante estimación del coeficiente alfa de Cronbach.

Los resultados se consideraron significativos con la p menor a 0,05.

## Resultados

Respondieron el cuestionario 918 estudiantes, de los cuales fueron descartadas 164 respuestas por contener menos del 80% de los datos. Se analizaron 854 respuestas, el 47% (401) tenía entre 21-24 años seguido del 33% (280) pertenecía al grupo de 17-20 años.

Las principales características de la muestra se detallan en la tabla 1. El consumo de alcohol y tabaco fue más frecuente en los estudiantes iniciales mientras que en los avanzados la marihuana y el modafinilo.

**Tabla N° 1:** Características de la muestra

	Estudiantes totales n (%)	Estudiantes iniciales, n (%)	Estudiantes avanzados, n (%)	OR y p
Número	854 (100)	537 (62,89)	317 (37,11)	
Sexo Femenino	618 (72,36)	397 (73,92)	221 (69,71)	
Masculino	231 (27,04)	136 (25,32)	95 (29,96)	
No binarios	6 (0,7)	5 (0,93)	1 (0,3)	
Realizan Actividad Física	555 (64,98)	338 (63,06)	217 (68,45)	OR 0.78 (0,58 – 1,01) p <0,11
Consumo de Alcohol	673 (78,80)	445 (83,02)	228 (71,92%)	OR 2,16 (1,54-3,04) p< 0,000008
Consumo de Marihuana	43 (5,03)	16 (2,99)	26 (8,2%)	OR 0,34 (0,8-0,65) p <0,0009
Modafinilo	4 (0,46)	3 (0,56)	30 (9,46%)	OR 0,67 (0,02-0,23) p< 0,00000001
Tabaquismo	52 (6,08)	22 (4,10)	1 (0,32%)	OR 9,2 (1,23-68) p <0,008
Origen;				
Córdoba capital	219 (25,64)	135 (25,13)	84 (26,5)	OR 0,77 (0,56-1,06) p <0,12
Córdoba interior	268 (31,38)	180 (33,51)	87 (27,4)	OR 1,33 (0,98-1,80) p <0,06
Otro país / provincia	367 (42,97)	221 (41,15)	146 (46,06)	OR 0,8 (0,61-1,08) p <0,17

Se puede observar en la tabla 2 la comparación entre las respuestas sobre calidad de vida, de las posibles respuestas se diferenciaron estadísticamente "bastante buena" en el grupo inicial y "regular", no encontrando diferencias en percepción de salud entre los grupos.

**Tabla N° 2:** Percepción de Calidad de vida y de salud en estudiantes de medicina de nivel inicial y avanzado

	Muestra total n y %	Estudiantes iniciales n y porcentaje	Estudiantes avanzados n y porcentaje	OR - p
Calidad de Vida				
Muy mala	13 (1,52)	5 (0,93)	8 (2,52)	OR 0,53 (0,17-1,64) p <0,27
Regular	155 (18,15)	91 (16,98)	64 (20,13)	OR 1,47 (1.02-2.11) p<0,03
Normal	306 (35,83)	190 (35,45)	116 (36,48)	OR 0,94 (0,71-1,26) p<0,76
Bastante buena	286 (33,49)	197 (36,57)	90 (28,30)	1,46 (1,08-1,97) p<0,01
Muy buena	94 (11,01)	54 (10,07)	40 (12,58)	0,77 (0,50-1,19) p=0,25
Percepción de Salud				
Muy insatisfecho	26 (3,04)	13 (2,43)	13 (4,09)	OR 0,58 (0,26-1,26) p=0,21
Un poco insatisfecho	228 (26,70)	137 (25,56)	91 (28,62)	OR 0,85 (0,62-1,16) p=0,33
Lo normal	281 (32,90)	179 (33,21)	103 (32,39)	OR 1,03 (0,77-1,93) p=0,82
Bastante satisfecho	239 (27,99)	154 (28,73)	85 (26,73)	OR 1,09 (0,80-1,49) p=0,58
Muy Satisfecho	80 (9,37)	54 (10,07)	26 (8,18)	OR 1,25 (0,76-2,04) p=0,39

Al agrupar CV normal, bastante buena y muy buena y compararla entre estudiantes avanzados e iniciales no se encontraron diferencias OR 1,2 (0,9-1,89) p<0,09, sin embargo, si se considera solo CV bastante buena y muy buena entre ambos grupos se obtiene un OR 1.65 (1.23-2.2) con una p<0.00058 a favor de la CV de los EI. Al tomar los peores puntajes, muy mala y regular, no hay diferencias estadísticamente significativas entre los grupos.

Al comparar entre los dos grupos las puntuaciones crudas medias del cuestionario y sus cuatro dominios tampoco se encontró diferencias significativas (Tablas 3 y 4)

**Tabla N° 3:** Cuestionario Whoqol Bref en Estudiantes Iniciales

Dominio	Media	Desvío estándar	Coeficiente de variación	Valor Mínimo	Valor Máximo	Amplitud
Físico	11,38	2,14	18,78	5,71	18,29	12,57
Psicológico	12,85	2,05	15,97	5,33	18	12,67
Relaciones sociales	12,88	3,18	24,73	4	20	16
Medio Ambiente	13,9	2,34	16,84	7	19,5	12,5
Autoevaluación de la Calidad de Vida	13,13	3,37	25,7	4	20	16
TOTAL	12,8	1,86	14,56	7,69	18	10,31

**Tabla N° 4:** Cuestionario Whoqol Bref en Estudiantes avanzados

Dominio	Media	Desviación Estándar	Coeficiente De Variación	Valor Mínimo	Valor Máximo	Amplitud
Físico	11,51	2,05	17,82	6,29	17,14	10,86
Psicológico	12,91	2,05	15,92	5,33	18	12,67
Relaciones Sociales	13,21	3,22	24,38	4	20	16
Medio Ambiente	13,73	2,67	19,45	5	18,5	13,5
Autoevaluación de la CV	12,69	3,61	28,4	4	20	16
TOTAL	12,81	1,97	15,37	6,31	16,77	10,46

En cuanto a la influencia de las variables estudiadas encontramos que mientras más positivas fueron las calificaciones en la CV, más satisfechos estaban con las distintas variables, autoevaluación de la CV, Salud Física, Salud Psicológica, Relaciones sociales y Entorno o medio ambiente. El alfa de Cronbach fue 0.8899.

Considerando toda la muestra, cuando se relacionó la CV con la edad, entre los que la calificaron como *muy mala*, 31% tenía entre 25-28 años; mientras que los que presentaron mayores calificaciones, tenían entre 21-24 años (p<0.0001). No fueron significativas las variables de género y la de región de procedencia (p: <0.07 y 0.52 respectivamente).

En cuanto a la actividad física en el grupo inicial quienes mejor describieron su calidad de vida (bastante buena y muy buena), realizaban actividad física, comparado con el resto que definió menor calidad de vida OR 1,95 (1,31-2,92) p <0,0009; al igual que en el grupo avanzado OR 4,13 (2,49-6,87) p <0,00000001.

Del grupo estudiantes avanzados que definieron su calidad de vida como bastante buena y muy buena (130), 98 tomaban alcohol, OR 0,6 (0,46-0,89), p <0,01; al igual que en el grupo de los EI, 230 personas OR 2,60 (1,51-4,45) p <0,0002.

## Discusión

Uno de los desafíos del uso del concepto de "calidad de vida" como base para nuestros resultados y medidas es que puede ser definido, y de igual manera medido en innumerables formas. Se ha asumido que estas medidas pueden ser influenciadas tanto por disciplinas académicas como por perspectivas ideológicas ^18^ .

Además, se ha demostrado que los estudiantes de medicina experimentan altos niveles de estrés durante el cursado de la carrera, lo cual puede tener efecto negativo en su esfera cognitiva y de aprendizaje. Hay estudios que sugieren que los trastornos en la salud mental empeoran luego de que los estudiantes comienzan la carrera y se mantienen durante todo el entrenamiento ^19^ .

Sumado a lo expresado anteriormente la pandemia Covid 19, a nivel mundial ha generado nuevas formas de aprendizaje e interrelaciones que podrían afectar la calidad de vida de las personas y de los estudiantes de medicina en particular, debido a la interacción que deben realizar con los pacientes sobre todo durante los últimos años de cursado.

Nuestro estudio está compuesto por una muestra de 854 estudiantes, con predominio de mujeres de 21 a 24 años, ha demostrado en general una buena Calidad de Vida, si consideramos el grupo que respondió normal a muy buena, lo cual es un dato de gran valor; sin embargo, si consideramos los mayores puntajes en CV, la misma es mejor en los Estudiantes iniciales. Este hallazgo es similar al de otros estudios en los que la CV es menor a medida que se avanza en la carrera como lo describió Irribarra en Chile con una muestra 411 estudiantes ^20^ , Chazán en Brasil ^21^ y Zhang en China con 1686 estudiantes de medicina ^22^ . En nuestro estudio no encontramos diferencias al considerar la percepción de salud ni los diferentes dominios en contraste con estudios citados anteriormente donde los varones perciben mejor su salud.

Contrariamente, hay investigaciones que, utilizando el instrumento WHOQOL-BREF, determinaron que la CV es invariable en la trayectoria académica. Malibary ^23^ en Arabia Saudita, con 630 estudiantes de Medicina, no encontró diferencias significativas en la CV entre los estudiantes de los distintos años, a pesar de que los mismos están expuestos a diferentes entornos de aprendizaje y cargas de trabajo a medida que avanzan en la carrera, al igual que en Egipto ^24^ Malibary propone que esto puede explicarse por el plan de estudios, señala los programas de aprendizaje basado en problemas (ABP); que son introducidos a principios del segundo año y corresponden a diferentes cursos integrados que enfocan de forma conjunta los aspectos básicos y clínicos de los temas, junto con una orientación temprana a la capacitación clínica durante los primeros 3 años de la escuela de Medicina). El ABP ayuda a los estudiantes a integrar su conocimiento científico básico con habilidades de diagnóstico y razonamiento clínico de forma temprana y progresiva. Esta situación explicaría la diferencia en la CV entre estudiantes que se relacionan con uno u otro plan de estudio.

Zhang, en la Facultad de Medicina de China, detalla cómo la carga horaria varía en los distintos niveles; además de que el plan de estudios de tercer año incluye muchos casos clínicos integrados, lo que requiere más horas de estudio ^22^ . Situación similar a la realidad de la Universidad Nacional de Córdoba, donde el plan de estudios de la Facultad de Ciencias Médicas aumenta la carga horaria a partir de 4to año con las materias clínicas y un abordaje integral de las materias correspondientes a los primeros años.

No encontramos diferencias significativas en calidad de vida al comparar géneros, lo cual difiere de lo descripto en Perú por Leitón Espinoza ^25^ , en Chile por dos estudios, uno de Irribarra ^20^ y otro de Delannays Hernandez ^7^ y Chazán en Brasil ^21^ . Este hallazgo es difícil de explicar, ya que existen informes en Argentina ^26^ de la mayor dificultad de las mujeres para acceder a la educación al contraer obligaciones de cuidados familiares antes que los varones y sobrecarga doméstica ^26^ . Sin embargo, a pesar de ser todos países de la región es posible que existan en este aspecto diferencias culturales que ameritarían un estudio antropológico y social más profundo.

La CV no estuvo influenciada por el origen de los estudiantes, lo cual contrasta con lo señalado por Irribarra, no hemos encontrado otros resultados en la literatura que evalúen este aspecto.

En cuanto a la realización de actividad física, si bien no fue cuantificada, los estudiantes de ambos grupos que afirmaron realizarla obtuvieron los mayores puntajes en CV, consistentemente a lo demostrado previamente por Irribarra, Oviedo en Ecuador(^27)^ y en Colombia por Barboza Granados ^28^ . Particularmente este último autor sostiene que, por un lado, se encontró que el nivel vigoroso de actividad física tiene un efecto favorable sobre la salud general; por el otro, que el nivel moderado tiene un efecto favorable para la salud mental. En ambas dimensiones, los resultados fueron significativos, en comparación con el nivel ligero. También se encontraron resultados positivos relacionados a la función social y a la vitalidad.

Fue llamativa la cantidad de estudiantes tanto iniciales como avanzados que consumen alcohol, en toda la muestra 78%. El 83% de los estudiantes iniciales lo hacen y si bien el porcentaje permaneció alto, en los EA disminuyó en forma significativa. Aunque en este estudio no se determinó el perfil de consumo de los estudiantes, nuestros hallazgos coinciden con Tarrant en el Reino Unido ^29^ .

Con respecto a los otros hábitos tóxicos, los EI consumen más tabaco, mientras los EA consumen más marihuana y modafinilo. Si bien no fueron porcentajes altos, el estudio no estuvo diseñado para determinar el policonsumo o relacionar el hábito tabáquico con el de fumar marihuana como lo describe Torres en Uruguay ^30^ .

La fortaleza de este estudio está conformada por el tamaño muestral, mientras que como debilidades señalamos algunos datos que no fueron recogidos para analizar como el estado civil, y las limitaciones propias del cuestionario en esta población particular, ya que no mide situaciones específicas como la relación con el cuerpo docente, la satisfacción por la elección de la carrera y la casa de estudios o experiencias en prácticas entre otras, así como también otra debilidad es la manera en que se recogieron los datos, si bien en forma anónima y a través de un sistema de la Facultad, los estudiantes con peor calidad de vida pudieron no haber contestado la encuesta. Creemos que este punto es importante y debe ser retomado en la presencialidad plena para corroborar los resultados que presentamos.

Los datos aportados son útiles para delinear propuestas que mejoren la calidad de vida en estudiantes universitarios de medicina por ejemplo a través de la actividad física y demostramos realidades que necesitan mayor análisis como el consumo de alcohol, marihuana, tabaco y modafinilo, para comprender estos fenómenos y proteger la salud de nuestros estudiantes más allá de la percepción que cada uno de ellos tenga de su calidad de vida.

## Conclusión

Si bien la CV es percibida predominantemente como normal en los estudiantes de Medicina de la Universidad Nacional de Córdoba, los mejores puntajes se observan en los estudiantes iniciales y en los que realizan actividad física, por lo tanto, se deberían delinear políticas universitarias en este sentido. Son necesarios más estudios que nos permitan comprender el consumo de sustancias tóxicas en esta población.
